# Debate: what is the best method to monitor surgical performance?

**DOI:** 10.1186/s12893-016-0131-8

**Published:** 2016-04-05

**Authors:** Stefan H. Steiner, William H. Woodall

**Affiliations:** Department of Statistic and Actuarial Science, University of Waterloo, Waterloo, ON N2L 3G1 Canada; Department of Statistics, Virginia Tech, Blacksburg, VA 24061-0439 USA

## Abstract

**Background:**

There is considerable recent interest in the monitoring of individual surgeon or hospital surgical outcomes. If one aggregates data over time and assesses performance with a funnel plot, then the detection of any process deterioration or improvement could be delayed. The variable life adjusted display (VLAD) is widely used for monitoring on a case-by-case basis, but we show that use of the risk-adjusted Bernoulli cumulative sum (RA-CUSUM) chart leads to much better performance.

**Discussion:**

We use simulation to illustrate that the RA-CUSUM chart has better performance than the VLAD in detecting changes in the rates of adverse events.

**Summary:**

We recommend the RA-CUSUM approach over the VLAD approach for monitoring surgical performance. If the VLAD is used, we recommend running the RA-CUSUM chart in the background to generate signals that the process performance has changed.

## Background

The recent article by O’Neill et al. [1] opened the debate about the best method for prospectively monitoring surgical performance. They described the variable life adjusted display (VLAD) approach, introduced by Lovegrove et al. [2] and Poloniecki et al. [3], and discussed its advantages and disadvantages. They made the point that retrospective monitoring, for example with funnel plots, may result in delayed reactions to worsening performance and requires the choice of an arbitrary time interval for the data aggregation. Alternatives to the VLAD chart that also allow real time prospective monitoring have been developed that alleviate some of its drawbacks. Here we describe the risk-adjusted cumulative sum (RA-CUSUM) chart [4] and discuss how the RA-CUSUM avoids the main drawbacks of the VLAD approach. We note that these methods can be used to monitor the rate of any adverse event or complication not just for mortality rates that we use for illustration.

## Discussion

The VLAD chart plots the cumulative sum of the expected minus observed mortality. Sustained increases or decreases suggest either better or worse performance than expected. While the VLAD appears popular and provides an easy-to-interpret visual display, there are a number of disadvantages and limitations of the VLAD approach, some of which are described in O’Neill et al. [1].

### Disadvantages of the VLAD

The VLAD approach does not easily lend itself to setting useful control limits. The control limits shown in O’Neill et al. [1] are always widening. If based on the true standard deviation of the VLAD statistic, the limits would not be wavy. As a result of the widening, they are unsuited for detecting changes in performance that might occur at times other than the start of the monitoring. The problem is that the VLAD chart can build up credit and be a long way from the relevant control limit when the performance changes, thus failing to quickly detect the change.

Also, we need to be careful in interpreting the control limits for the VLAD presented in O’Neill et al. [1] as 5 % limits. With a VLAD chart, as with any monitoring method that accumulates evidence across time, the chance of a signal at time *t* depends on the level of the cumulative statistic at time *t*-1. If the cumulative value is close to one of the control limits it is more likely that a signal will arise than if the value is near the middle of the control limits.

For continuous monitoring approaches that accumulate evidence across multiple patients it is better to assess performance in terms of the run length distribution, where run length is defined as the number of patients until the chart statistic exceeds the control limits. Often the performance is summarized by the average run length (ARL) and we want a large ARL value when in-control, i.e., when the mortality rate is as expected, and a small ARL value when the actual mortality rate is substantially greater (or less) than the expected rate.

### Risk adjusted cumulative sum (RA-CUSUM) approach

An alternative prospective monitoring approach, first proposed by Steiner et al. [4], is the RA-CUSUM chart based on

1 $$ {X}_t= \max \left(0,\kern0.5em {X}_{t-1}+{m}_t\right),\kern0.5em t=1,2,\dots $$

where the score (based on the likelihood ratio) for each patient in (1) is given by

2 $$ {m}_t=\left\{\begin{array}{l} \log \left[1/\left(1-{p}_t+R{p}_t\right)\right]\kern2em \mathrm{if}\ \mathrm{patient}\ t\ \mathrm{survives}\\ {} \log \left[R/\left(1-{p}_t+R{p}_t\right)\right]\kern1.22em \mathrm{if}\ \mathrm{patient}\ t\ \mathrm{dies}\end{array}\right. $$

*X*_0_ = 0 and *p*_*t*_ is the expected probability of death for patient *t*. To detect an increased death rate we select *R* > 1. Note that in contrast to the expected-observed scores used in the VLAD, the patient scores given by (2) are positive when a death occurs and negative for a success. The cumulative sum *X*_*t*_ accumulates evidence of poor performance over time, it is never allowed to be negative so that a deterioration of performance at any time (even after a series of favourable results immediately before the deterioration) will be quickly detected. The patient scores given by (2) are optimal [5], in terms of *average* run length, to compare the hypotheses

3 $$ \begin{array}{l}{H}_0:\ \mathrm{o}\mathrm{dds}\ \mathrm{o}\mathrm{f}\ \mathrm{death}\ \mathrm{f}\mathrm{o}\mathrm{r}\ \mathrm{patient}\kern0.5em t = {p}_t/\left(1-{p}_t\right)\\ {}{H}_A:\ \mathrm{o}\mathrm{dds}\ \mathrm{o}\mathrm{f}\ \mathrm{death}\ \mathrm{f}\mathrm{o}\mathrm{r}\ \mathrm{patient}\kern0.5em t = R{p}_t/\left(1-{p}_t\right)\end{array} $$

repeatedly over time provided all patients have the same risk, and likely close to optimal otherwise. Note that under *H*_0_ the odds of death equals what is expected, while *H*_*A*_ corresponds to a change in performance. We choose *R* based on the size of the process change we are interested in quickly identifying with *R* = 2 a common choice. The hypotheses are set up in terms of the odds of death rather than the probability of death for mathematical convenience and to prevent any probability from exceeding one.

**Fig. 1:**
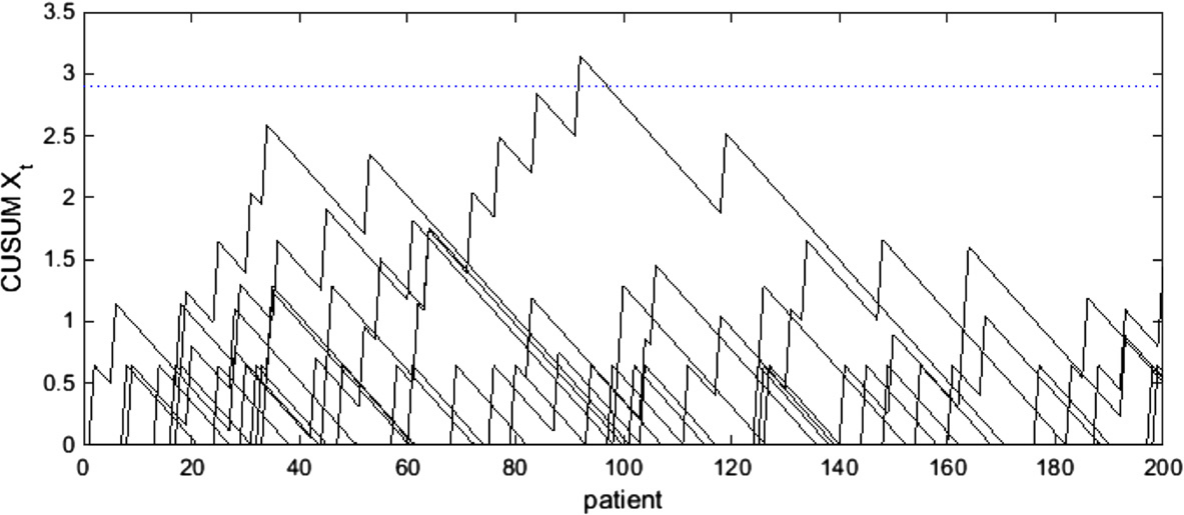
10 example RA-CUSUMs showing 10 simulated surgeons with control limit at 2.9

We use the RA-CUSUM to signal evidence that the mortality rate has increased when *X*_*t*_ exceeds a horizontal control limit. Setting an appropriate control limit for a RA-CUSUM based on a desired in-control average run length is discussed in Steiner et al. [4] and Zhang and Woodall [6]. Figure 1 provides 10 example RA-CUSUMs corresponding to 10 different simulated surgeons when the observed and expected mortality rates are 5 % and the control limit is set at 2.9, which gives an in-control ARL of roughly 1300 patients. Note that each upward jump in the CUSUM statistic corresponds to a death while decreases correspond to a string of successes.

### Comparison of RA-CUSUM and VLAD approaches

To compare the performance of the RA-CUSUM and the VLAD charts we consider the four scenarios presented in Fig. 1 of O’Neill et al. [1]. While these scenarios do not require risk adjustment since all patients are assumed to be interchangeable, they still provide a useful comparison. We discuss the use of risk adjustment later in this paper. We set up the RA-CUSUM chart with *R* = 2 and a control limit so that the in-control ARL is roughly 1300 patients. This resulted in control limits of 2.9, 1.7, 3.4 and 3.4 in the four scenarios respectively. In all scenarios we simulated 10,000 RA-CUSUM charts and determined the proportion of the CUSUM statistics that exceeded the control limit at 10, 25, 50, 75, 100, 150 and 200 patients. Note that we are considering the proportion of charts whose RA-CUSUM statistic exceeds the control limit *at* a given patient not *by* a given patient. Also, the simulated RA-CUSUM charts were not reset after exceeding the control limit to match the usual application of a VLAD chart. The results for the VLAD approach in Table 1 were taken from Fig. 1 in O’Neill et al. [1] since they conducted the same simulation using the VLAD approach. The bolded values in Table 1 correspond to better performance.

**Table 1:** Simulation comparison of VLAD and RA-CUSUM approaches

Scenario: expected, actual mortality rate	Method	Proportion of chart exceeding the control limit at patient						
		10	25	50	75	100	150	200
1: 5 %, 5 %	VLAD	0.01	0.02	0.02	0.02	0.03	0.02	0.03
	RA-CUSUM	**0.00**	**0.00**	**0.01**	**0.01**	**0.02**	**0.02**	0.03
2: 1 %, 2 %	VLAD	**0.01**	**0.08**	**0.08**	0.06	0.14	0.18	0.21
	RA-CUSUM	0.00	0.01	0.05	**0.08**	**0.15**	**0.22**	**0.32**
3: 10 %, 10 % but with 6 deaths in a row patients 94–99	VLAD	0.02	0.02	0.02	0.02	0.32	**0.01**	**0.01**
	RA-CUSUM	**0.00**	**0.00**	**0.00**	**0.01**	**1.00**	0.37	0.22
4: 10 %, 10 % for first 100 patients, then 12.5 %	VLAD	0.01	0.01	0.02	0.02	0.02	0.06	0.07
	RA-CUSUM	**0.00**	**0.00**	**0.01**	**0.01**	**0.01**	**0.07**	**0.12**

The bolded values correspond to better performance

In Scenario 1 where we do not want a signal, since the actual surgical performance matches the expected, the VLAD and RA-CUSUM charts give similar results. For Scenario 2 while the VLAD has a greater chance of detecting the change in 25 or 50 patients the RA-CUSUM is superior for 75+ patients. Due to the building up of credit problem with the VLAD discussed earlier, the performance of the VLAD will on average be much worse than the RA-CUSUM if the change from 1 to 2 % mortality occurs sometime after the start of the monitoring. The same problem illustrated in Scenario 3 where the 6 deaths in a row generate a signal 100 % of the time by the RA-CUSUM but only 32 % of the time by the VLAD. Similarly, Scenario 4 results show the RA-CUSUM is more likely to detect the small change in mortality rate that starts at patient 100. Note that here we designed the RA-CUSUM to be optimal for detecting a doubling of the odds of death (corresponding to the mortality rate of about 18 %). Had we instead designed the RA-CUSUM to detect an increase to 12.5 % (odds ratio of about 1.3) the RA-CUSUM chart would have signaled in 15 % of the cases by 200 patients.

The RA-CUSUM charts we illustrate here are designed to detect only increases in the mortality rate. It is straightforward to run two RA-CUSUMs simultaneously to look for both increases and decreases in the mortality rate – see Steiner et al. [4] and Steiner [7] for details. Also, usually a signal on a RA-CUSUM chart would result in an investigation into the cause and the chart would be reset to start at zero. Recall that the RA-CUSUM charts simulated to obtain Table 1 were not reset after a signal. In scenario 3, had we reset the RA-CUSUM after the signal at 100 patients the chance of a further signal for the remaining patients would have been very small.

### Risk adjustment

In most surgical monitoring applications risk adjustment is required since patients are typically heterogeneous. Our simulation did not use risk adjustment only to simplify the comparison of the VLAD and RA-CUSUM charts and to match the scenarios considered by O’Neill [1]. In practice the risk adjustment is provided by a risk model that summarizes how patient characteristics, such as age, sex, etc., affect the chance of mortality or other adverse event. In application the risk model is established before starting the monitoring and can come from the published literature or from a prior study conducted in the context of the application.

For the RA-CUSUM the risk model is used to give *p*_*t*_ in (2) and thus, together with the observed outcome, affects the score given to the *t*^th^ patient. With a VLAD chart the risk model provides the expected mortality or adverse event rate. While comparing these two different approaches to adjust for patient risk was not the focus of this article, it is also of interest. The results in Moustakides [5] suggest that the RA-CUSUM approach is close to optimal for changes in the mortality rate to that given by the alternative hypothesis in (3). Steiner et al. [4] provided a comparison of scores based on (2) and expected minus observed scores (matching the VLAD approach) in a specific context. This comparison suggests that the scores based on (2) are better in all cases except when the change in mortality rate is very small.

## Summary and Conclusions

The prospective monitoring of surgical performance can contribute to improved outcomes and should be promoted. For the reasons we explained, the RA-CUSUM method has superior performance to the VLAD approach. However, because the VLAD is easy to interpretable and perhaps familiar to some surgeons, we suggest showing the VLAD with signals generated by a RA-CUSUM chart run in the background. As pointed out by Woodall et al. [8], this is the recommendation also made by Sherlaw-Johnson [9], one of the originators of the VLAD.
